# Supplementary material for: Kupffer cells prevent pancreatic ductal adenocarcinoma metastasis to the liver in mice
Source: Nat Commun. 2023 Oct 10;14:6330. doi: 10.1038/s41467-023-41771-z (PMC10564762; doi:10.1038/s41467-023-41771-z)
Supplement: Supplementary file 1 — Supplementary Information [file 41467_2023_41771_MOESM1_ESM.pdf]

Supplementary material

Kupffer cells prevent pancreatic ductal adenocarcinoma metastasis to the liver in mice

Authors: Stacy K. Thomas[1,2]; Max M. Wattenberg[1,2]; Shaanti Choi-Bose[1,2]; Mark Uhlik[3,‡]; Ben Harrison[3]; Heather Coho[1,2]; Christopher R. Cassella[1,2]; Meredith L. Stone[1,2]; Dhruv Patel[1,2]; Kelly Markowitz[1,2]; Devora Delman[1,2]; Michael Chisamore[4]; Jeremy Drees[3]; Nandita Bose[3]; Gregory L. Beatty[1,2,*]

Affiliations:
[1]Division of Hematology-Oncology, Department of Medicine, Perelman School of Medicine, University of Pennsylvania, Philadelphia, PA
[2]Abramson Cancer Center, Perelman School of Medicine, University of Pennsylvania, Philadelphia, PA
[3]HiberCell Inc, Roseville, MN
[4]Merck & Co., Inc., Kenilworth, NJ, USA
‡Currently at OncXerna, Waltham, MA
*Corresponding author.

Corresponding author:
Gregory L. Beatty, MD, PhD, Abramson Cancer Center of the University of Pennsylvania, Perelman Center for Advanced Medicine, South Pavilion Rm 8-107, 3400 Civic Center Blvd. Bldg 421, Philadelphia, PA 19104-5156. Tele: 215-746-7764. Email: gregory.beatty@pennmedicine.upenn.edu

Supplementary Figure 1

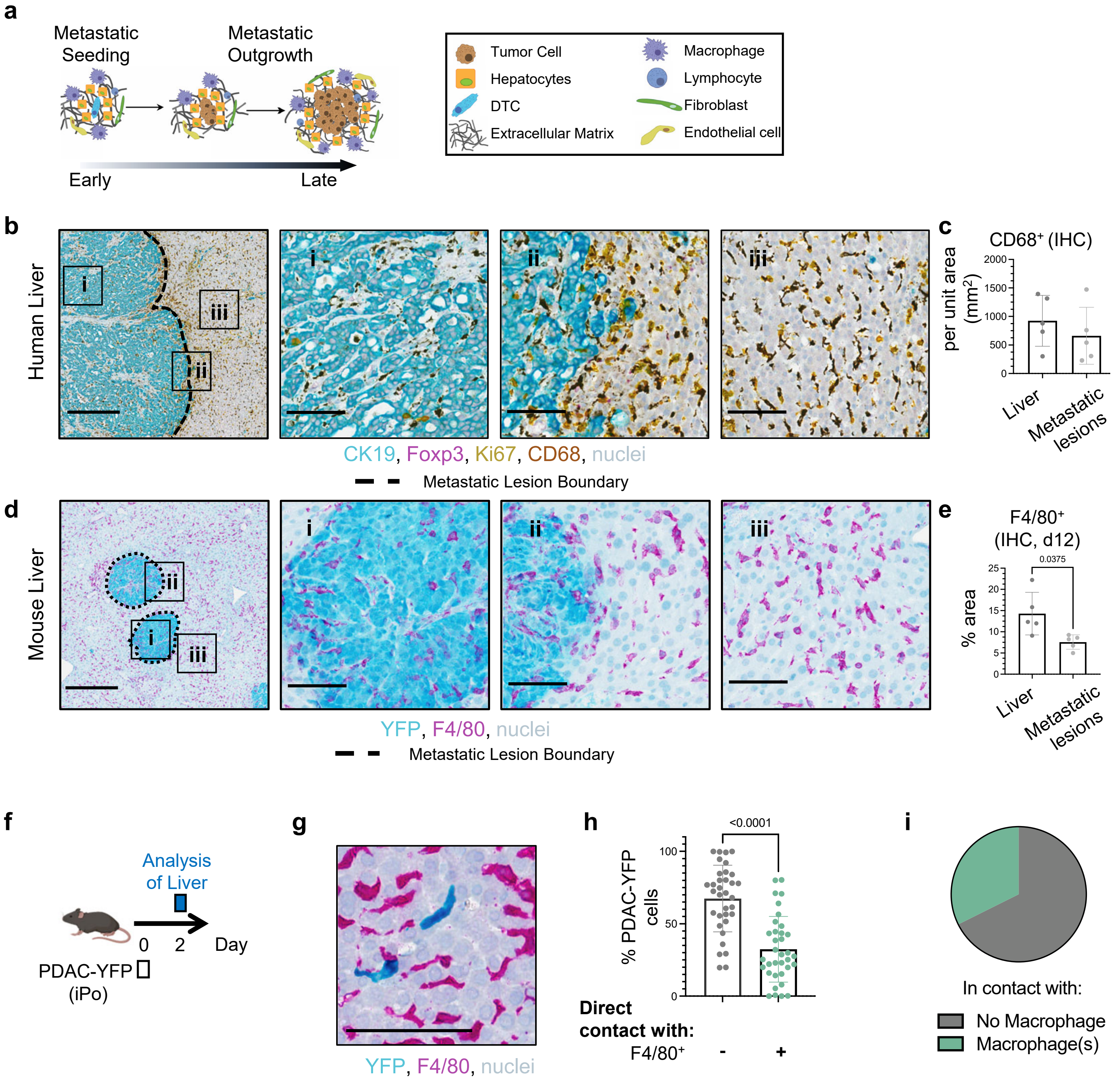

Supplementary Figure 1 | Liver macrophages engage with disseminated tumor cells in mice and humans. **a**, Schema showing metastatic process in distant tissues. **b**, Representative images of a human PDAC liver metastasis biopsy stained using multiplex immunohistochemistry (mIHC) to detect CK19 (teal), Foxp3 (purple), Ki67 (yellow), CD68 (brown), and nuclei (blue, hematoxylin). Scale bar, 500μm. Insets show (i) metastatic lesion, (ii) peripheral margin of lesion, and (iii) normal adjacent liver tissue. Scale bar, 100μm. Metastatic lesions, black dashed lines. **c**, Quantification of CD68+ macrophages per area of indicated region in human liver metastases. Data are representative of n=5 biopsies. **d**, Representative images of livers from treatment-naive mice at 12 days after intraportal (iPo) injection of 500,000 PDAC-YFP cells. Tissues are stained using mIHC to detect YFP+ tumor cells (teal), F4/80+ liver macrophages (purple), and nuclei (blue, hematoxylin). Scale bar, 500μm. Insets show (i) metastatic lesion, (ii) peripheral margin of lesion, and (iii) normal adjacent liver tissue. Scale bar, 100μm. **e**, Quantification of F4/80+ macrophages shown as percent area of indicated region (parenchyma adjacent liver or metastatic lesion) in liver of untreated mice at 12 days after iPo injection of PDAC-YFP cells. Data are representative of n=5 livers. **f**, Study design for g-i. **g**, Representative image of a liver stained using mIHC to detect YFP+ tumor cells (teal), F4/80 (purple), and nuclei (blue, hematoxylin) on Day 2. Scale bar, 100μm. **h**, Quantification of cell-cell interactions between seeding tumor cells and F4/80+ liver macrophages in mouse livers (n=35) stained as in h. **i**, Pie chart. P values determined by unpaired two-tailed Welch's t test. Mean ± SD is shown. iPo, intraportal.

Supplementary Figure 2

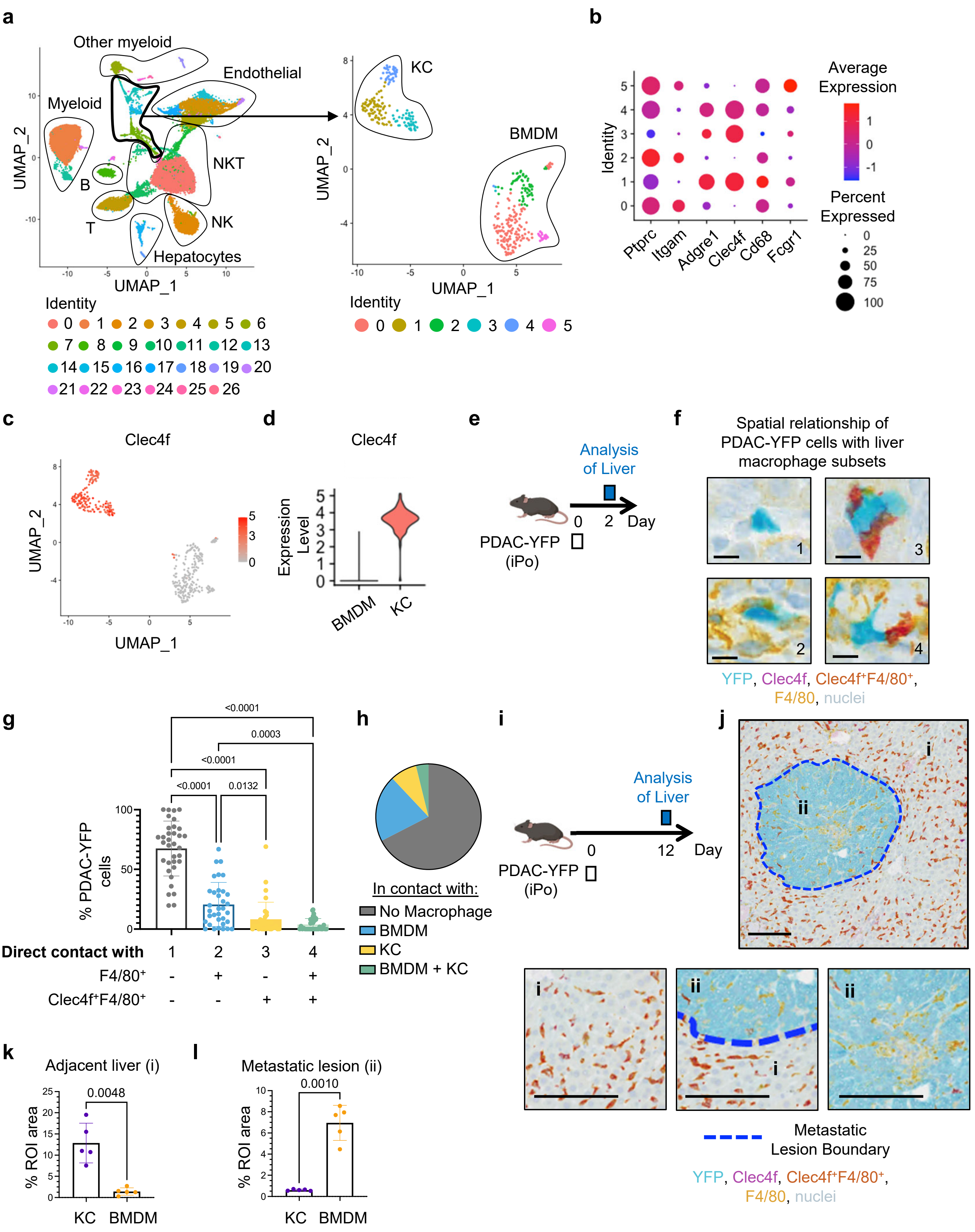

Supplementary Figure 2 | Liver macrophage subsets exist in distinct spatial niches in the setting of liver metastasis. a, UMAP analysis of 27 distinct cell clusters isolated from four normal C57BL/6 mouse livers (left). UMAP visualization of 6 identified macrophage clusters (right). **b,** Dot plot of key cellular markers used to identify macrophage populations. Dot color represents the average expression, and size indicates percent expression. **c,** Feature plot of *Clec4f* expression among macrophage populations. **d,** Violin plot of normalized *Clec4f* expression in indicated macrophage sub-populations. **e,** Study design for **f-h**. Mice received an iPo injection of 200,000 PDAC-YFP tumor cells on day 0. On Day 2, mice were euthanized, and livers analyzed. **f,** Representative images of livers stained using multiplex immunohistochemistry (mIHC) to detect YFP^{+} tumor cells (teal), Clec4f (purple) and F4/80 (yellow) at 2 days after iPo injection of PDAC-YFP tumor cells. Images depict a seeding tumor cell in contact with (1) no macrophage, (2) a BMDM, (3) a KC and (4) both a BMDM and a KC. Scale bars, 10μm. **g,** Quantification of cell-cell interactions between seeding tumor cells and liver macrophages in mouse livers (n=35). **h,** Pie chart summarizing data shown in **g**. **i,** Study design for **j-l**. C57L/6 mice received an iPo injection of 500,000 PDAC-YFP tumor cells on Day 0. On Day 12, mice were euthanized, and livers analyzed. **j,** Representative image of a liver stained using mIHC to detect YFP (teal), Clec4f (purple) and F4/80 (yellow) at 12 days after iPo injection of PDAC-YFP tumor cells. Scale bar, 200μm. Bottom row shows regions of liver parenchyma (i) and the metastatic lesion (ii) **k-l,** Quantification of $Clec4f^{+}F4/80^{+}$ KCs and $Clec4f^{-}F4/80^{+}$ BMDMs shown as percent area of the liver parenchyma (region i, **k**) or metastatic lesion (region ii, **l**) in livers of untreated mice (n=5) at 12 days after iPo injection of PDAC-YFP cells. *P* values determined by unpaired two-tailed Welch's t test and one-way ANOVA with Tukey's test. Mean $\pm$ SD is shown. BMDMs, bone marrow derived macrophages; iPo, intraportal; KCs, Kupffer cells.

Supplementary Figure 3

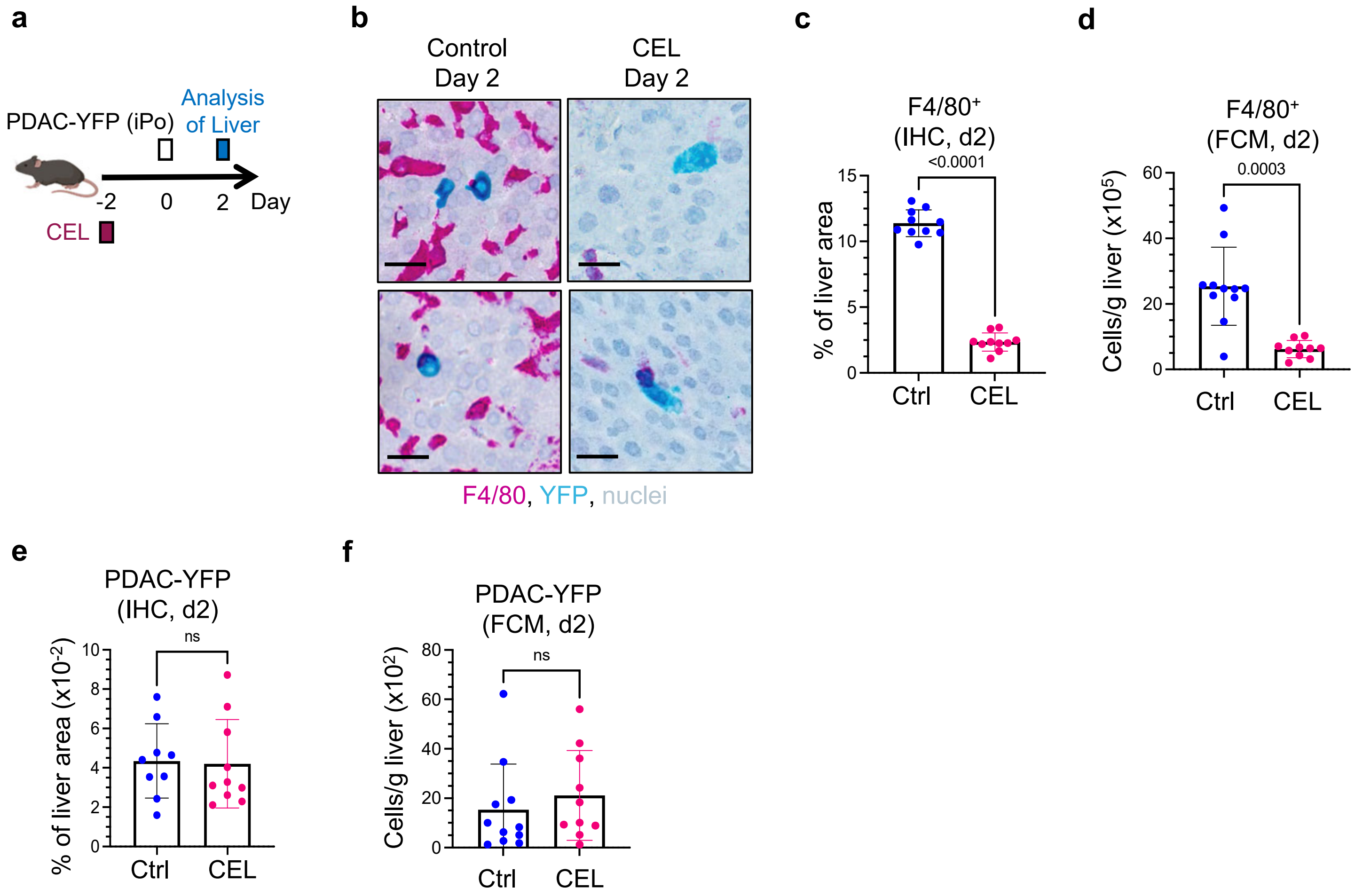

Supplementary Figure 3 | Metastatic seeding is independent of liver macrophages. a, Study design **b-f**. **b**, Representative images of seeding tumor cells in a control (left) and CEL treated (right) liver stained using mIHC to detect YFP (teal) and F4/80 (purple) on Day 2. Scale bar, 25µm. **c,** Quantification of F4/80$^+$ macrophages shown as percent area in livers of mice (n=10 per group) at 2 days after iPo injection of PDAC-YFP cells. **d,** Frequency of F4/80$^+$ cells detected by FCM in liver on Day 2. **e**, PDAC-YFP (YFP$^+$) tumor cells detected by IHC in liver on Day 2 and shown as percent of liver area. **f**, Quantification of PDAC-YFP tumor cells detected in the liver on Day 2 (d2) by flow cytometry (FCM). *P* values determined by unpaired two-tailed Welch's t test. Data are representative of n=5 independent experiments. Mean $\pm$ SD is shown. FCM, flow cytometry; IHC, immunohistochemistry; CEL, clodronate encapsulated liposomes.

Supplementary Figure 4

a.

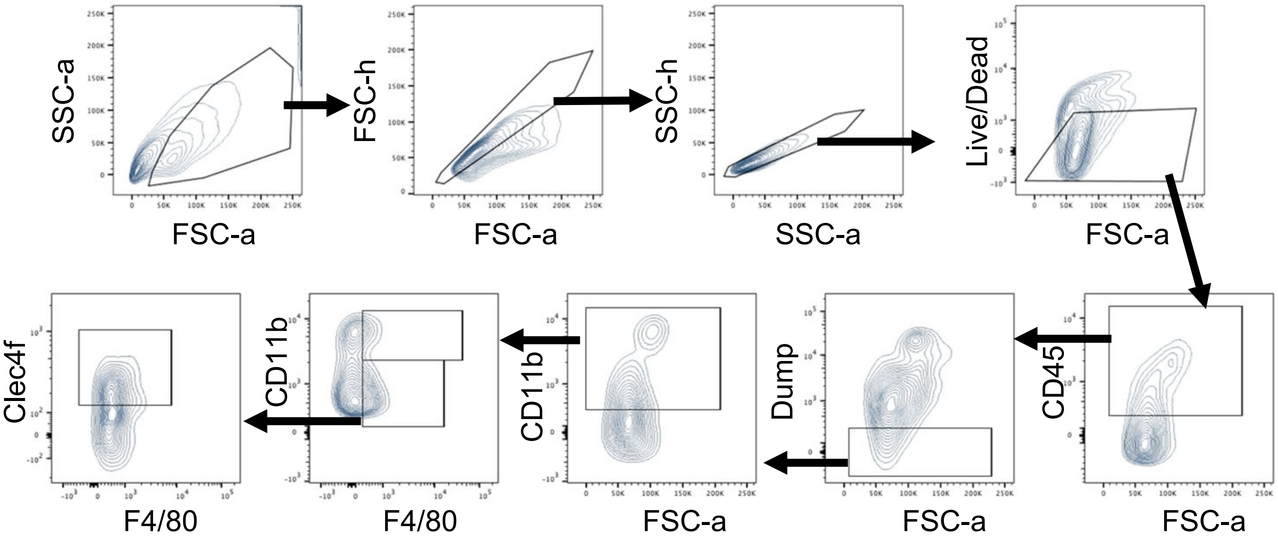

b.

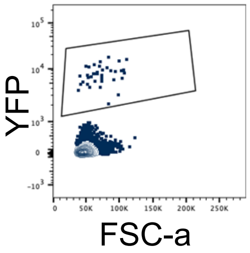

Supplementary Figure 4 | Flow Cytometry gating schema. a, Representative dot plots showing gating strategy for identifying macrophage populations in the liver. Gates were based on FMOs. Macrophages were defined as live, single cells, expressing CD45, CD11b, and F4/80 and lacking expression of CD3, CD19, CD11c, and Ly6G. Kupffer cells (KC) were distinguished from bone marrow derived macrophages (BMDM) based on Clec4f expression. Staining is representative of a liver sample from a C57BL/6 mouse. **b,** Representative FCM gating strategies for detection of YFP^{+} tumor cells in the liver after iPo injection of PDAC-YFP cells. Populations shown were gated on single, live cells.

Supplementary Figure 5

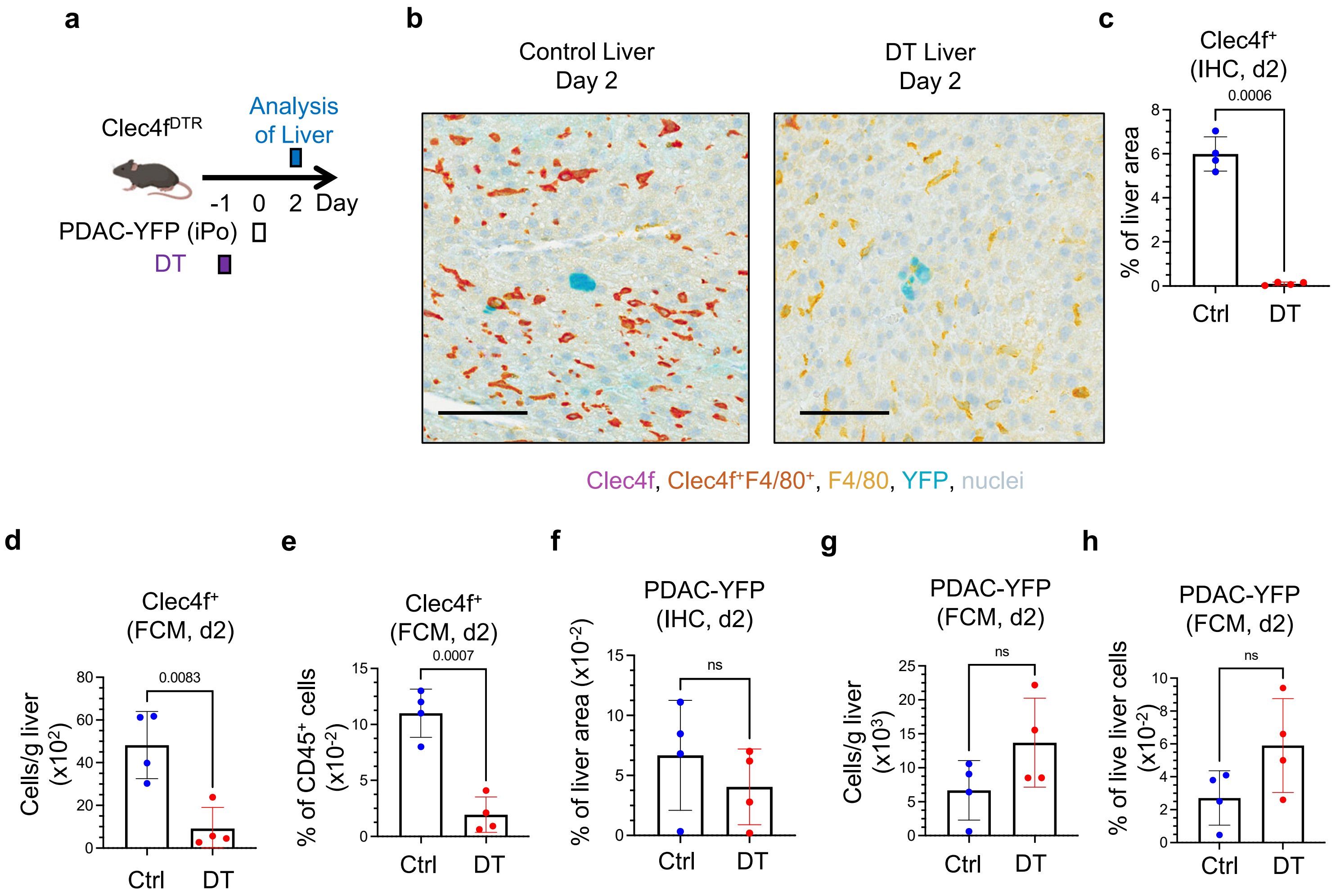

Supplementary Figure 5 | Metastatic seeding in the liver is independent of Kupffer cells. a, Study design for **b-h.** Clec4f^{DTR} mice were treated without (Ctrl) or with diphtheria toxin (DT) to deplete Kupffer Cells. On Day 0, mice received an iPo injection of 200,000 PDAC-YFP tumor cells. Mice were euthanized and livers analyzed on Day 2. Data are representative of n=2 experiments. **b,** Representative images of control (left) and DT treated (right) liver stained using multiplex IHC to detect YFP$^+$ tumor cells (teal), Clec4f (purple) and F4/80 (yellow) at 2 days after iPo injection of PDAC-YFP cells. Scale bar, 100µm. **c,** Quantification of Clec4f$^+$ KCs shown as percent area in livers of mice (n=4 per group) at 2 days after iPo injection of PDAC-YFP cells. **d-e,** Quantification of cell counts per gram liver (**d**) and frequency (**e**) of Clec4f$^+$ KCs detected by FCM in liver on Day 2. **f,** Quantification of YFP$^+$ tumor cells shown as percent area in livers of mice at 2 days after iPo injection of PDAC-YFP cells. **g-h,** Quantification of cell counts per gram liver (**g**) and frequency (**h**) of PDAC-YFP tumor cells detected by FCM in liver on Day 2. *P* values determined by unpaired two-tailed Welch's t test. Data are representative of n=2 independent experiments. Mean $\pm$ SD is shown. FCM, flow cytometry; IHC, immunohistochemistry; iPo, intraportal; DT, Diphtheria toxin.

Supplementary Figure 6

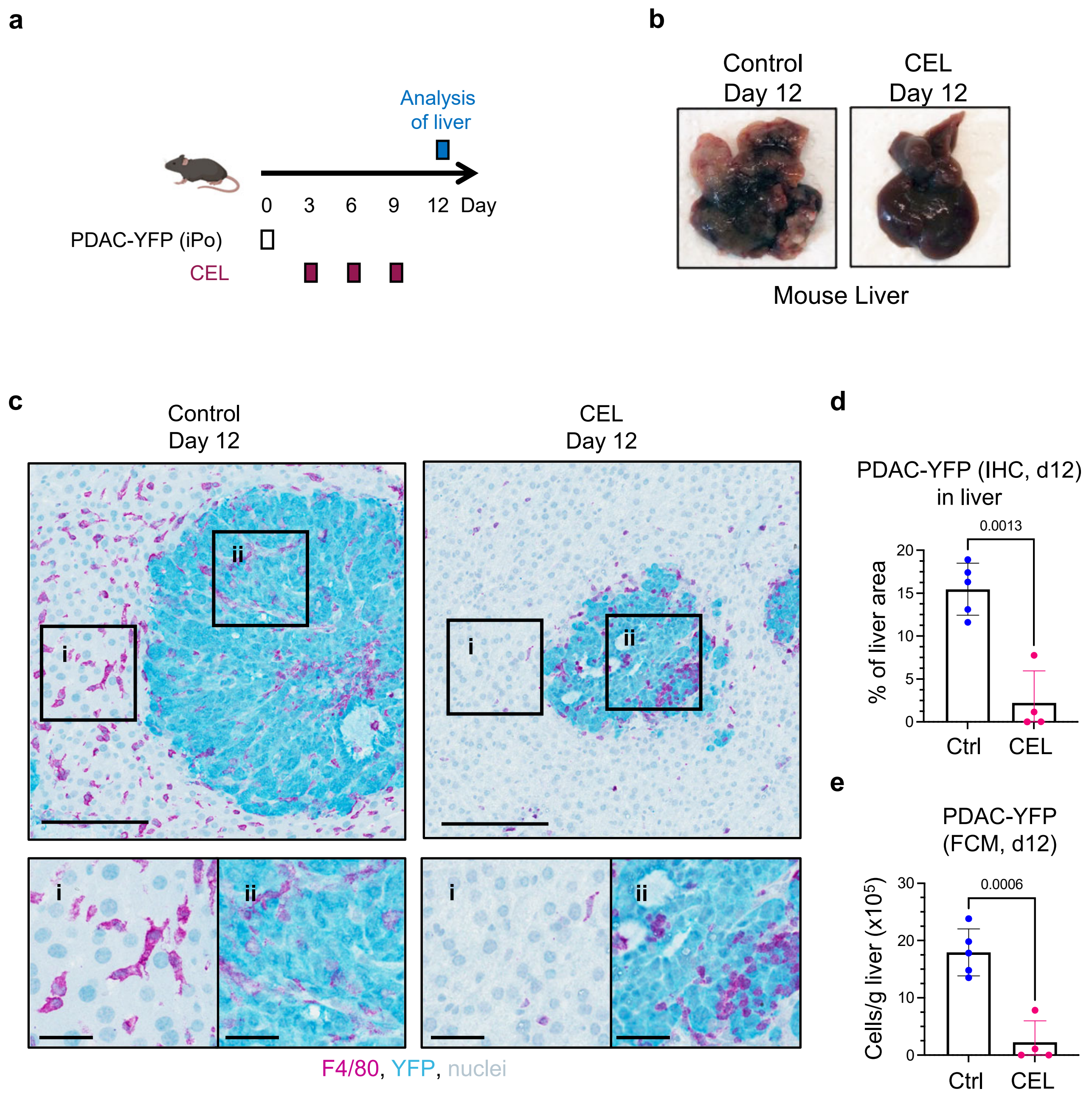

Supplementary Figure 6 | Liver macrophages promote metastatic outgrowth. a, Study design for **b-e**. Data are representative of n=2 experiments. **b**, Representative gross images of livers from mice at 12 days after iPo injection of PDAC-YFP cells. **c**, Representative images of a control (left) and CEL treated (right) liver stained using multiplex IHC to detect YFP (teal) and F4/80 (purple) on Day 12 after iPo injection of PDAC-YFP tumor cells. i, Adjacent liver tissue. ii, Metastatic lesion. Top row images, scale bar, 200μm. Bottom row images, scale bar, 50μm. **d**, Quantification of PDAC-YFP (YFP^+) tumor cells detected by IHC in liver on Day 12 and shown as percent of liver area. **e**, Quantification of PDAC-YFP tumor cells detected in the liver (n=5 in Ctrl group, n=4 in CEL group) on Day 12 (d12) by FCM. P values determined by unpaired two-tailed Welch's t test. Data are representative of n=2 independent experiments. Mean ± SD is shown. CEL, clodronate encapsulated liposomes; FCM, flow cytometry; IHC, immunohistochemistry; iPo, intraportal.

Supplementary Figure 7

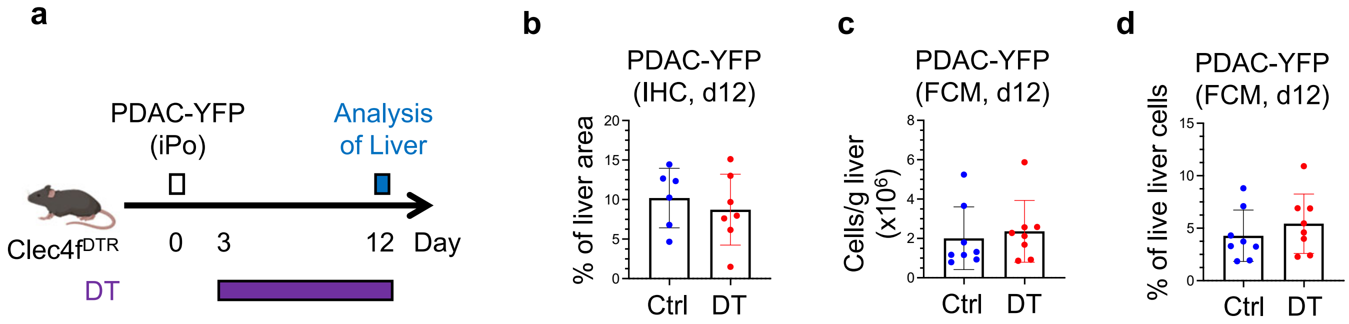

Supplementary Figure 7 | Effect of daily Kupffer cell depletion on metastatic outgrowth in the liver. a, Study design for **b-d**. Clec4f^{DTR} mice received an iPo injection of 500,000 PDAC-YFP tumor cells on Day 0. Mice were treated without (Ctrl) or with diphtheria toxin (DT) daily beginning on Day 3 to deplete Kupffer cells. On Day 12, mice were euthanized, and livers analyzed. Data are representative of n=3 experiments. **b-c,** Quantification of YFP^{+} tumor cells using **(b)** multiplex IHC and **(c)** FCM in livers of mice (n=8 per group) at 12 days after iPo injection of PDAC-YFP cells. **d,** Frequency of PDAC-YFP tumor cells detected by FCM in liver on Day 12. *P* values determined by unpaired two-tailed Welch's t test. Data are representative of n=3 independent experiments. Mean $\pm$ SD is shown. FCM, flow cytometry; IHC, immunohistochemistry; iPo, intraportal; DT, Diphtheria toxin.

Supplementary Figure 8

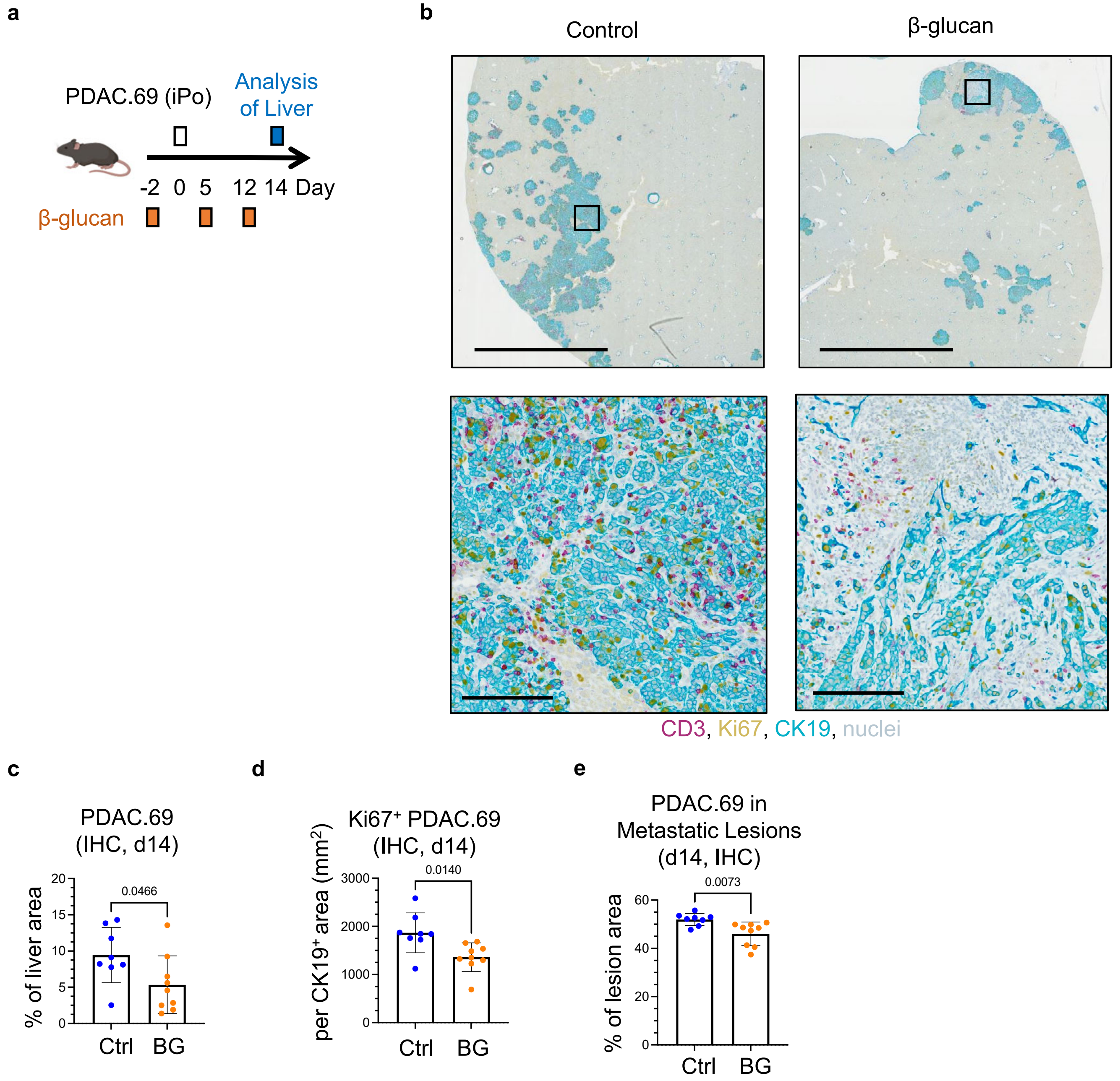

Supplementary Figure 8 | β-Glucan treatment inhibits PDAC.69 metastatic growth in the liver. a, Study design for **b-e**. Mice were treated without (Ctrl) or with β-Glucan (BG). Weekly dosing of BG was initiated on Day -2. On Day 0, mice received an iPo injection of 500,000 PDAC.69 tumor cells. On Day 14, mice were euthanized, and livers analyzed. Data are representative of n=2 experiments. **b,** Representative images of livers from control and β-Glucan treated mice at 14 days after iPo injection of PDAC.69 cells. Tissues stained using multiplex IHC to detect $CK19^+$ tumor cells (teal), CD3 (purple) and Ki67 (yellow). Nuclei (blue) were stained with hematoxylin. Top row images, scale bar, 5mm. Bottom row images showing metastatic lesions, scale bar, 200μm. **c,** PDAC.69 ($CK19^+$) tumor cells detected by IHC in liver (n=8 in Ctrl group, n=9 in BG group) on Day 14 and shown as percent of liver area. **d,** $CK19^+Ki67^+$ PDAC-YFP tumor cells detected by IHC in liver on Day 14 and shown as cells per $CK19^+$ area (mm^2). **e,** PDAC.69 ($CK19^+$) tumor cells detected by IHC in liver metastatic lesions on Day 14 and shown as percent of metastatic lesion area as drawn in **Fig. 2h**. *P* values determined by unpaired two-tailed Welch's t test. Data are representative of n=2 independent experiments. IHC, immunohistochemistry; iPo, intraportal.

Supplementary Figure 9

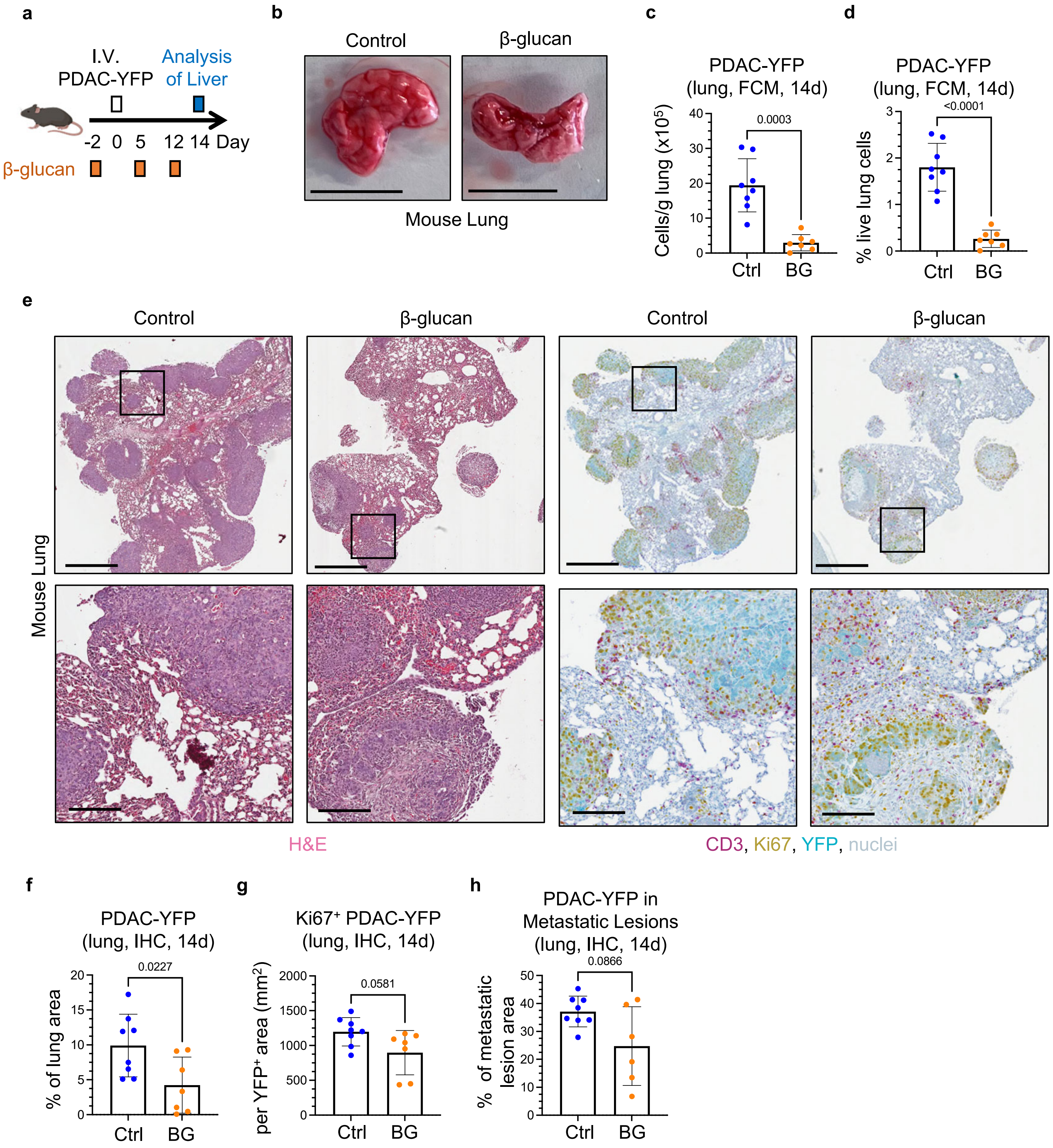

Supplementary Figure 9 | β-Glucan treatment inhibits PDAC metastasis in the lung. a, Study design for **b-h**. Mice were treated without (Ctrl) or with β-Glucan (BG). Weekly dosing of β-glucan was initiated on Day -2. On Day 0, mice received an IV injection of 500,000 PDAC-YFP tumor cells. On Day 14 (d14), mice were euthanized, and lungs analyzed. Data are representative of n=2 experiments. **b,** Representative gross images of dissected lungs from mice at 14 days after IV injection of PDAC-YFP cells. Scale bar, 1cm. **c,** Quantification of cell counts per gram lung and **d,** frequency of PDAC-YFP tumor cells detected by FCM in lung (n=8 in Ctrl group, n=7 in BG group) on Day 14. **e,** Representative images of lungs from control and BG treated mice at 14 days after IV injection of PDAC-YFP cells. Tissues stained using hematoxylin and eosin (H&E) (left) and mIHC (right) to detect YFP (teal), CD3 (purple) and Ki67 (yellow). Nuclei (blue) are stained with hematoxylin. Top row images, scale bar, 1mm. Bottom row images, scale bar, 200μm. **f,** PDAC-YFP (YFP^+) tumor cells detected by IHC in lung on Day 14 and shown as percent of lung area. **g,** YFP^+Ki67^+ PDAC-YFP tumor cells detected by IHC in lung on Day 14 and shown as cells per YFP^+ area (mm^2). **h,** PDAC-YFP (YFP^+) tumor cells detected by IHC in lung metastatic lesions on Day 14 and shown as percent of metastatic lesion area. *P* values determined by unpaired two-tailed Welch's t test. Data are representative of n=4 independent experiments. FCM, flow cytometry; IHC, immunohistochemistry; IV, intravenous; Ctrl, control; BG, β-glucan.

Supplementary Figure 10

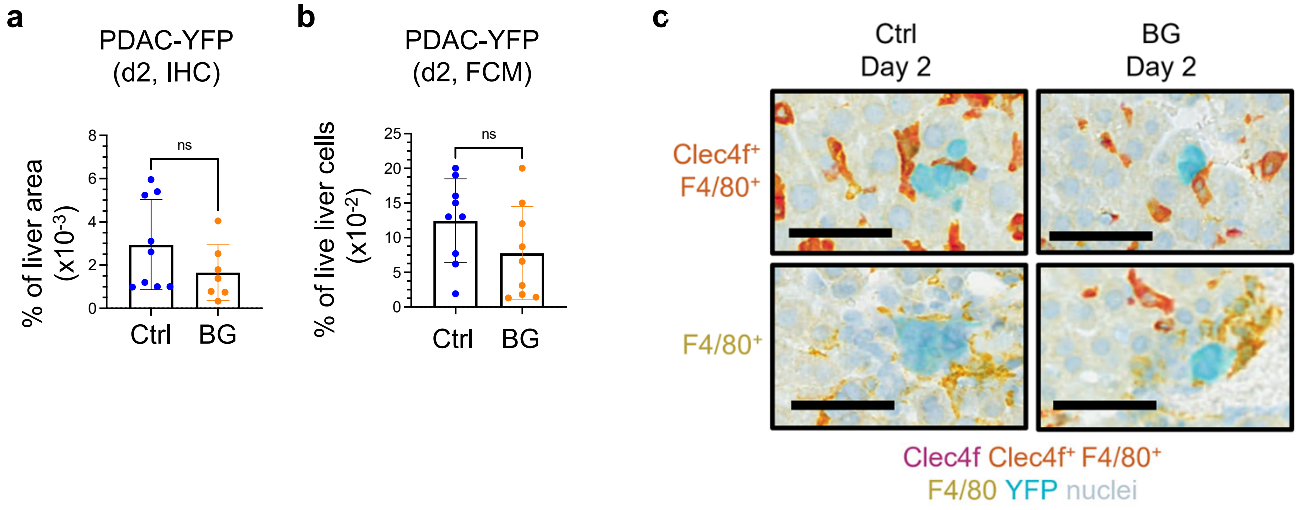

Supplementary Figure 10 | Metastatic seeding is not impacted by β-Glucan treatment. Study design described in **Fig. 1k**. Briefly, mice (n=9 per group) were treated on day -2 without (Ctrl) or with β-glucan (BG). On Day 0, mice received an iPo injection of 200,000 PDAC-YFP tumor cells. On Day 2, mice were euthanized, and livers analyzed. **a**, Quantification of YFP+ tumor cells shown as percent area in livers at 2 days after iPo injection of PDAC-YFP cells. **b**, Frequency of PDAC-YFP tumor cells detected by FCM in liver on Day 2. **c**, Representative images of seeding tumor cells interacting with liver macrophages in livers of control and BG-treated mice at 2 days after iPo injection of PDAC-YFP cells. Tissues were stained using mIHC to detect YFP (teal), Clec4f (purple), F4/80 (yellow), and nuclei (blue, hematoxylin). Scale bar, 50µm. P values determined by unpaired two-tailed Welch's t test. Data are representative of n=4 independent experiments. Mean + SD is shown. FCM, flow cytometry; IHC, immunohistochemistry; Ctrl, control; BG, β-glucan.

Supplementary Figure 11

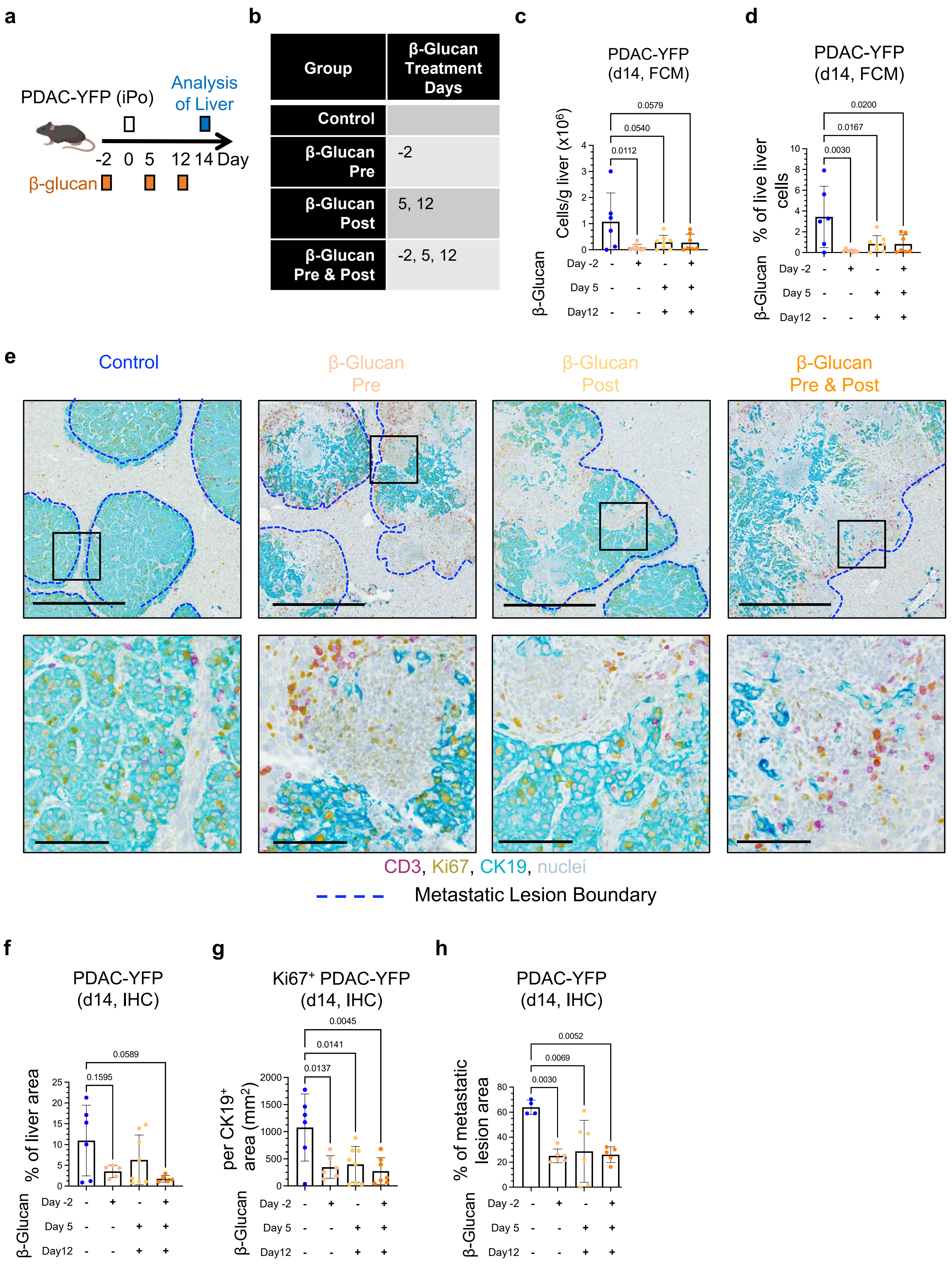

Supplementary Figure 11 | β-Glucan (BG) treatment delivered before or after metastatic challenge inhibits liver metastasis. a, Study design for **b-h.** Four cohorts of mice were studied. Briefly, on Day 0, all mice received an iPo injection of 200,000 PDAC-YFP tumor cells. BG was dosed as indicated in **b**. On Day 14, mice were euthanized, and livers analyzed. **c,** Quantification of cell counts per gram liver and **d,** frequency of PDAC-YFP cells detected by FCM in liver (n=6 in Ctrl group, n=7 in BG pre and BG pre+post groups, n=8 in BG post group) on Day 14. **e,** Representative images of livers from control (Ctrl) and β-Glucan treated mice at 14 days after iPo injection of PDAC cells. Tissues stained using multiplex IHC to detect CK19 (teal), CD3 (purple) and Ki67 (yellow). Nuclei (blue) are stained with hematoxylin. Top row images, scale bar, 500μm. Bottom row images of metastatic lesion, scale bar, 100μm. **f,** PDAC-YFP ($CK19^+$) tumor cells detected by IHC in liver on Day 14 and shown as percent of liver area. **g,** $CK19^+Ki67^+$ PDAC-YFP tumor cells detected by IHC in liver on Day 14 and shown as cells per $CK19^+$ area (mm^2). **h,** PDAC-YFP ($CK19^+$) tumor cells detected by IHC in liver metastatic lesions on Day 14 and shown as percent of metastatic lesion area. *P* values determined by one-way ANOVA with Tukey's test. Data are representative of n=3 independent experiments. Mean $\pm$ SD is shown. FCM, flow cytometry; IHC, immunohistochemistry.

Supplementary Figure 12

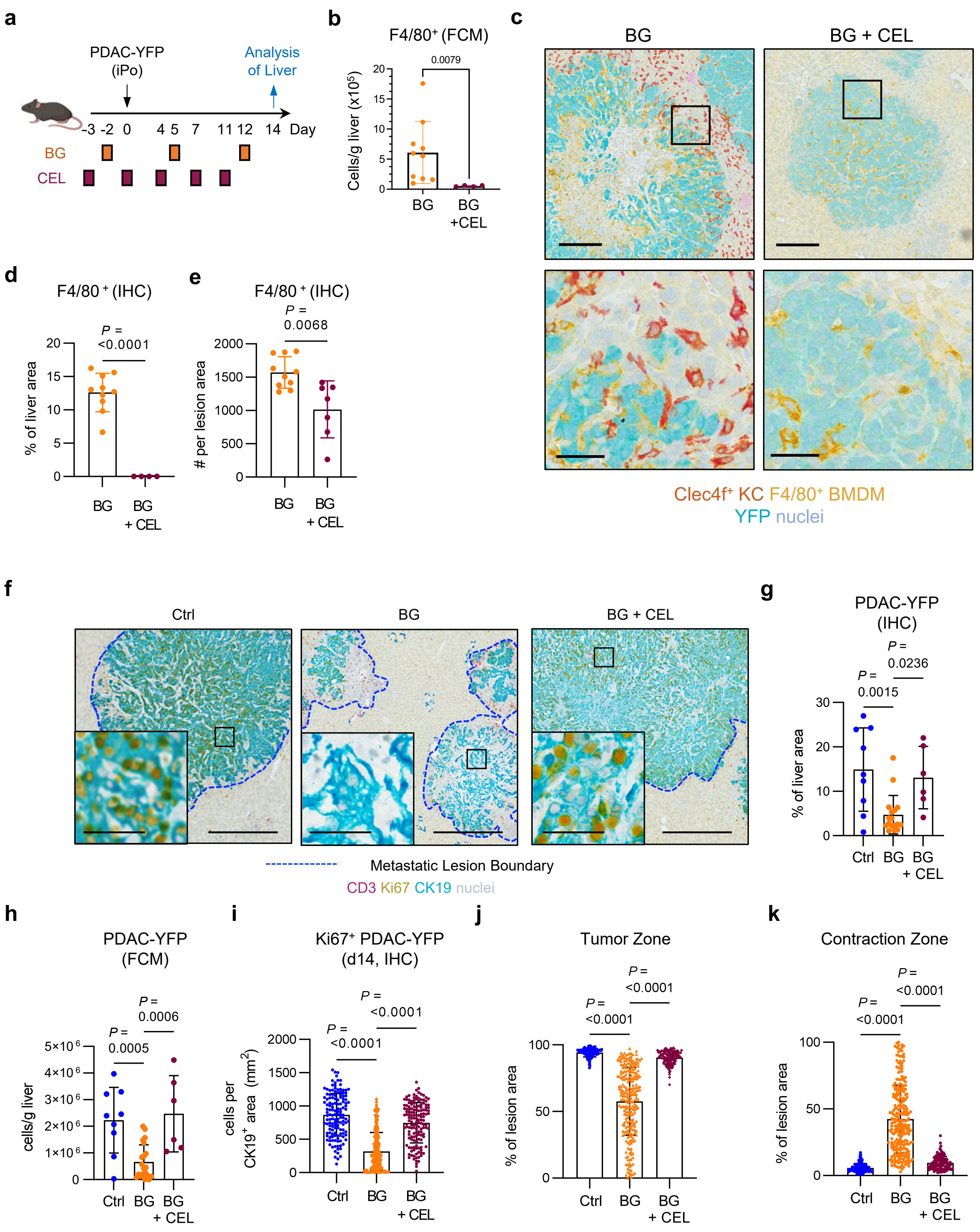

Supplementary Figure 12 | β-Glucan (BG) anti-metastatic activity requires liver macrophages. a, Study design for **b-k.** Mice were treated with clodronate encapsulated liposomes (CEL) to deplete bone marrow derived macrophages (BMDMs) every 3-4 days starting on day -3. Mice were then treated with weekly doses of β-glucan beginning on Day -2. On Day 0, mice received an iPo injection of 200,000 PDAC-YFP tumor cells. On Day 14, mice were euthanized, and livers analyzed. **b,** Quantification of F4/80$^+$ BMDMs detected by FCM in liver (n=10 in BG group, n=7 in BG+CEL group) on day 14. **c,** Representative images of control (left) and CEL treated (right) liver stained using multiplex IHC to detect YFP$^+$ tumor cells (teal), Clec4f (purple) and F4/80 (yellow) at 14 days after iPo injection of PDAC-YFP cells. Scale bar, 200µm. Higher magnification scale bar, 40µm. **d-e,** Quantification of YFP$^+$ tumor cells shown as percent of liver area (**d**) and lesions area (**e**) in livers of mice at 14 days after iPo injection of PDAC-YFP cells. *P* values determined by unpaired two-tailed Welch's t test (d) and Mann Whitney U test (e). **f,** Representative images of control (left), β-glucan (middle) and β-glucan + CEL (right) treated liver stained using multiplex IHC to detect YFP$^+$ tumor cells (teal), CD3 (purple), Ki67 (yellow) and nuclei (blue, hematoxylin). Inset shows metastatic lesion. Blue dotted line indicates metastatic lesions. Scale bar, 500µm. Inset scale bar, 50µm. **g,** Quantification of YFP$^+$ tumor cells shown as percent area in livers of mice (n=9 in Ctrl group, n=17 in BG group, n=6 in BG+CEL group at 14 days after iPo injection of PDAC-YFP cells. *P* values one-way ANOVA with Sidak's test **h,** Quantification of cell counts per gram liver of PDAC-YFP tumor cells detected by FCM in liver on Day 14. *P* values one-way ANOVA with Sidak's test **i,** CK19$^+$Ki67$^+$ PDAC-YFP tumor cells detected by IHC in liver on Day 14 and shown as cells per CK19$^+$ area (mm^2). Individual control (n=138), BG (n=260) and BG+CEL (n=145) tumor lesions were analyzed. *P* values determined by Kruskal-Wallis test with Dunn's multiple comparisons. **j-k**, Quantification of tumor zone (**j**) and contraction zone (**k**) in individual control (n=138), BG (n=260) and BG+CEL (n=145) tumor lesions on day 14 and shown as percent of lesion area as drawn in **Fig. 5c** in control mice or mice treated with BG+CEL. "Tumor zones" indicate histologically defined regions of metastatic lesions with dense CK19$^+$ cell staining and "contraction zones" indicate regions with decreased CK19$^+$ tumor cell density. *P* values determined by Kruskal-Wallis test with Dunn's multiple comparisons. Data are representative of n=2 independent experiments. Mean $\pm$ SD is shown. FCM, flow cytometry; BG, β-glucan; CEL, clodronate encapsulated liposomes; IHC, immunohistochemistry.

Supplementary Figure 13

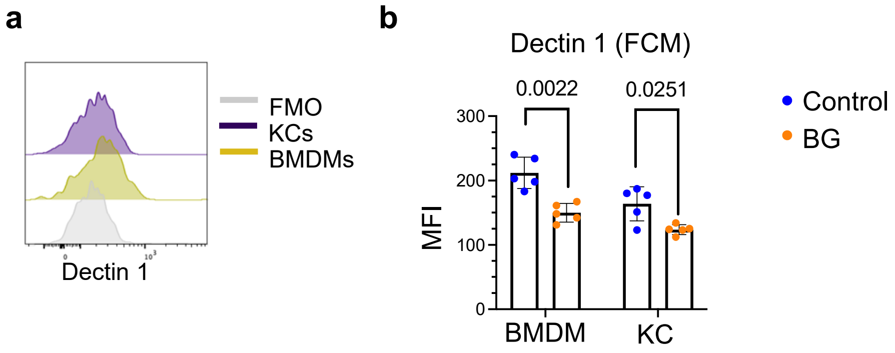

Supplementary Figure 13 | β-Glucan receptor, Dectin 1 (Clec7a), expression by liver macrophage subsets. a, Histogram of expression of the pattern recognition receptor, Dectin 1, on murine liver macrophage subsets by FCM. **b ,** Study design described in **Fig. 3a**. Shown is mean fluorescence intensity (MFI) of Dectin 1 expression detected by FCM on liver macrophages in livers of control or β-Glucan (BG) treated mice (n=5 per group) on Day 2 after BG treatment. *P* values determined by Welch's t Test. Data are representative of n=2 independent experiments. Mean $\pm$ SD is shown. FCM, flow cytometry; KC, Kupffer cell; BMDM, bone marrow derived macrophage.

Supplementary Figure 14

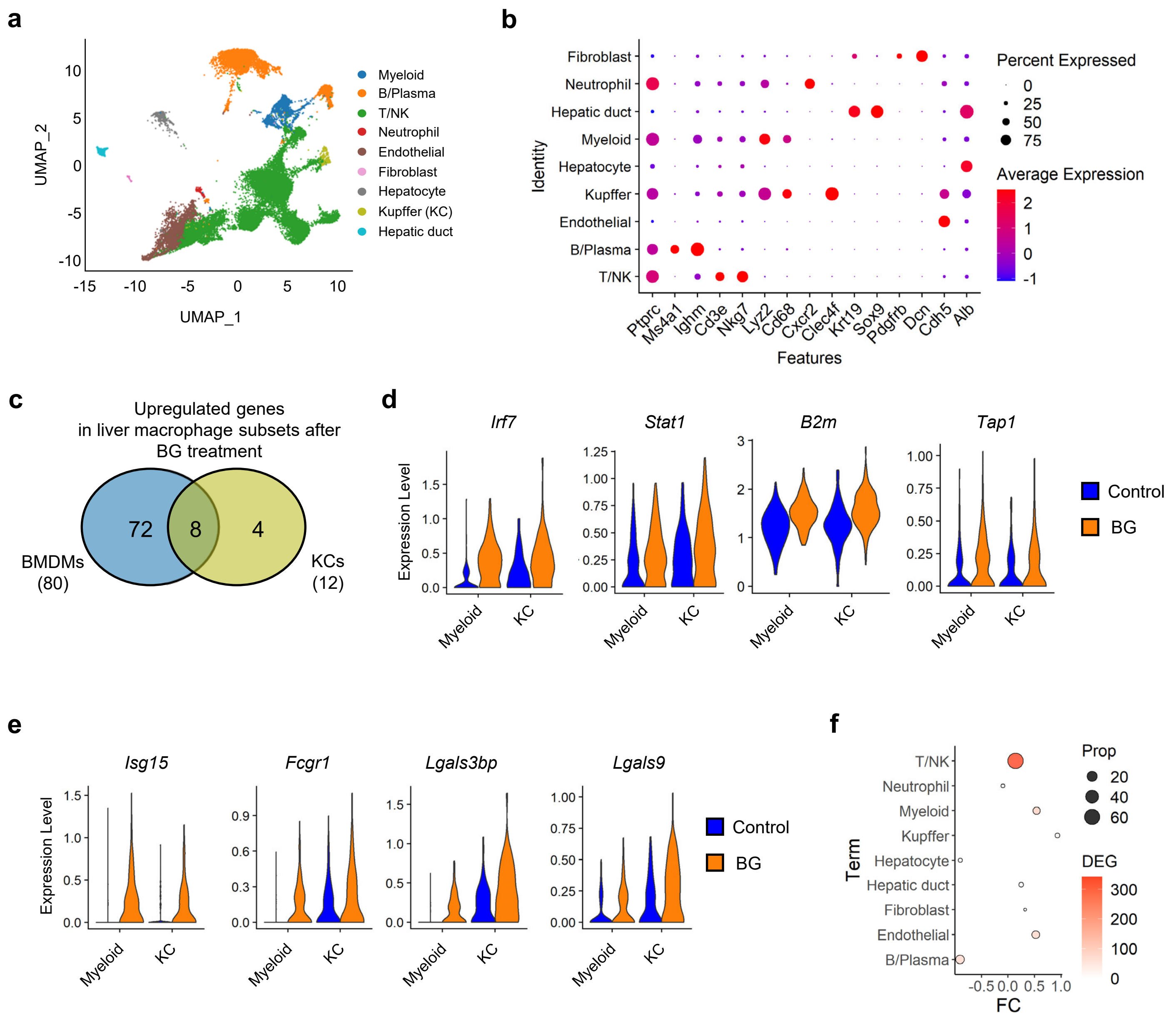

Supplementary Figure 14 | β-Glucan (BG) treatment triggers early remodeling of macrophage and T cell transcriptional programs. a, UMAP of liver cells defined by single cell RNA sequencing (scRNAseq). Study design as in **Fig. 3a**. Briefly, livers were isolated from control mice or mice treated two days prior with β-Glucan. **b,** Dotplot of lineage markers used to define liver cell populations. **c,** Venn diagram of differentially expressed genes in bone marrow derived macrophages (BMDMs) and Kupffer cells (KCs) in β-Glucan treated livers as compared to control livers. **d-e,** Violin plots of selected genes upregulated in BMDMs and/or KCs after β-Glucan (orange) treatment versus control (blue). **f,** Dot plot of Fold-Change in liver cell population frequency in BG treated livers compared to control livers. Size represents proportion of total cells and color indicates number of differentially expressed genes in BG versus control. BG, β-Glucan; BMDM; bone marrow derived macrophage; KC, Kupffer cell; FC, fold change; DEG, differentially expressed gene; Prop; proportion.

Supplementary Figure 15

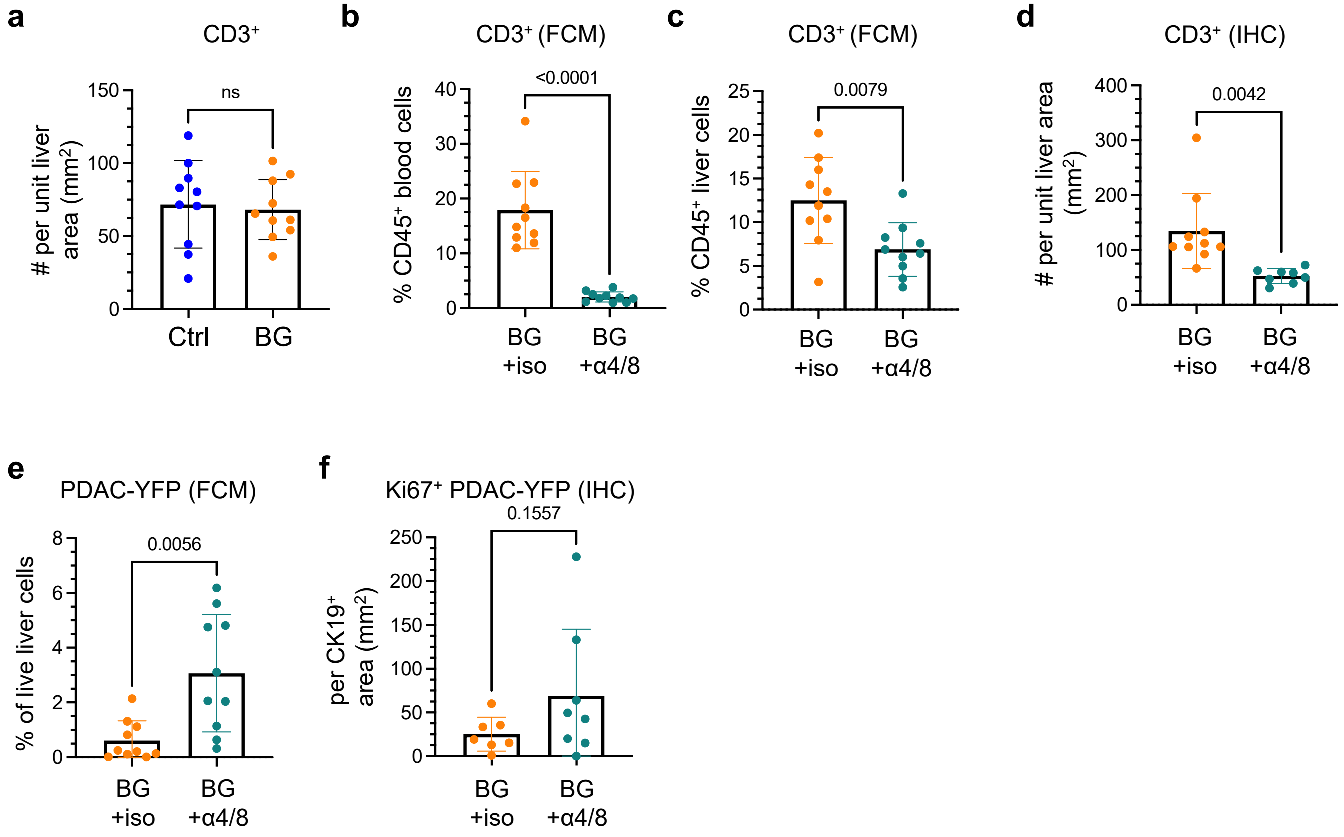

Supplementary Figure 15 | β-Glucan treatment stimulates T cell mediated anti-tumor immunity. a, $CD3^+$ T cells detected by IHC in livers of mice on day 14 after treatment as indicated. Study design described in **Fig. 1c**. Data are representative of n=4 independent experiments. **b-g,** Study design described in **Fig. 5f**. Briefly, mice were treated with isotype control or anti-CD8/anti-CD4 depleting antibodies every 3-4 days beginning on Day -3 to deplete T cells. Mice were then treated with weekly dosing of β-glucan beginning on Day -2. On Day 0, mice received an iPo injection of 200,000 PDAC-YFP tumor cells. On Day 14, mice were euthanized, and livers analyzed. Frequency of $CD3^+$ cells detected by FCM in (**b**) peripheral blood and (**c**) liver on Day 14. **d,** $CD3^+$ T cells detected by IHC in livers of mice on Day 14 and shown as cells per liver area (mm^2). **e,** Frequency of PDAC-YFP tumor cells detected by FCM in liver on Day 14. **f,** $CK19^+Ki67^+$ PDAC-YFP tumor cells detected by IHC in liver on Day 14 and shown as cells per $CK19^+$ area (mm^2). *P* values determined by unpaired two-tailed Welch's t test. Data are representative of n=3 independent experiments. Mean ± SD is shown. FCM, flow cytometry; IHC, immunohistochemistry; Ctrl, control; BG, β-glucan.

Table S1. ***In vivo*** **agents.**

Reagent	Clone	Dose	Vendor	Catalog #
**Diphtheria Toxin**		200ng	322326-1MG	Millipore Sigma
**Clodronate Liposomes**		200µL	Liposoma	C-010
**anti-PD-1**	RMP1-14	0.2mg	Bio X Cell	BE0146
**anti-CD4**	GK1.5	0.2mg	Bio X Cell	BP0003-1
**anti-CD8**	2.43	0.2mg	Bio X Cell	BE0061
**Rat IgG2b isotype control**	LFT-2	0.2mg	Bio X Cell	BE0090
**Odetiglucan**		1.2mg	Hibercell	

Table S2. Immunohistochemistry antibodies.

Application	Target Antigen	Target Species	Host Species	Clone	Dilution	Vendor	Catalog #
**IHC**	Clec4f	Mouse	Goat	Polyclonal	1:3000	R&D Systems	AF2784
**IHC**	GFP	Species Independent	Goat	Polyclonal	1:300	Abcam	ab6673
**IHC**	CD3	Mouse	Rabbit	Polyclonal	1:200	Abcam	ab5690
**IHC**	F4/80	Mouse	Rabbit	D2S9R	1:250	Cell Signaling	70076
**IHC**	Ki67	Mouse	Rabbit	D3B5	1:200	Cell Signaling	12202
**IHC**	CK19	Mouse	Rabbit	EPNCIR127B	1:800	Abcam	ab133496
**IHC**	CD68	Human	Mouse	KP1	1 drop	Roche	790-2931
**IHC**	Ki67	Human	Rabbit	30-9	1 drop	Roche	790-4286
**IHC**	CK19	Human	Mouse	A53-B/A2.26	1 drop	Roche	760-4281
**IHC**	Foxp3	Human	Mouse	236A/E7	1:25	Abcam	Ab20034

Table S3. Immunofluorescence antibodies.

Application	Target Antigen	Target Species	Host Species	Clone	Conjugation	Vendor	Catalog #	Dilution
**IF**	Cytokeratin	human	Mouse	AE1/AE3	Opal 480 or 780	Dako	M3515	1:50
**IF**	CD8a	human	Mouse	AMC908	Opal 520	Thermo	14-008-82	1:100
**IF**	Granzyme B	human	Rabbit	polyclonal	Opal 570	Abcam	4059	1:500
**IF**	FoxP3	human	Mouse	eBio7979	Opal 620	Thermo	14-7979-82	1:100
**IF**	CD4	human	Mouse	N1UG0	Opal 690	Thermo	14-2444-82	1:100
**IF**	Ki-67	human	Rabbit	SP6	Opal 780	Thermo	MA5-14520	1:50
**IF**	Melanoma Triple Cocktail	human	Mouse	polyclonal	Opal 480 or 780	Ventana	790-4677	1:5
**IF**	PD-L1	human	Rabbit	E1L3N	Opal 620	Cell Signaling	13684	1:100
**IF**	CD163	human	Rabbit	EPR19518	Opal 570	Abcam	182422	1:200
**IF**	CD80	human	Mouse	2E5	Opal 520	Genetex	GTX84700	1:200
**IF**	CD68	human	Mouse	KP1	Opal 480	Abcam	955	1:50
**IF**	CD206	human	Rabbit	polyclonal	Opal 690	Abcam	64693	1:1000
**IF**	Anti-Rabbit	Rabbit	Goat	polyclonal	HRP	Jackson Immunoresearch	111-035-144	1:100
**IF**	Anti-Mouse	Mouse	Goat	polyclonal	HRP	Jackson Immunoresearch	115-035-146	1:100

Table S4. Flow cytometry antibodies.

Application	Target Antigen	Target Species	Host Species	Clone	Conjugation	Dilution	Vendor	Catalog #
**FCM**	CD3	Mouse	Rat	17A2	Pacific Blue	1:100	Biolegend	100214
**FCM**	Ly6G	Mouse	Rat	1A8	Pacific Blue	1:100	Biolegend	127612
**FCM**	Ly6G	Mouse	Rat	1A8	BV 421	1:200	Biolegend	127627
**FCM**	CD11c	Mouse	Hamster	N418	Pacific Blue	1:100	Biolegend	117322
**FCM**	CD19	Mouse	Rat	6D5	Pacific Blue	1:100	Biolegend	115523
**FCM**	CD206	Mouse	Rat	C068C2	FITC	1:100	Biolegend	141704
**FCM**	CD38	Mouse	Rat	90	FITC	1:100	Biolegend	102705
**FCM**	CD19	Mouse	Rat	6D5	FITC	1:100	Biolegend	115506
**FCM**	Dectin1	Mouse	Rat	RH1	PE	1:100	Biolegend	144303
**FCM**	MHCI (H-2Kb/H-2Db)	Mouse	Mouse	28-8-6	PE	1:100	Biolegend	114607
**FCM**	CD38	Mouse	Rat	90	PE	1:100	Biolegend	102708
**FCM**	CD11c	Mouse	Hamster	HL3	PE	1:100	Biolegend	553802
**FCM**	CD206	Mouse	Rat	C068C2	PE	1:100	Biolegend	141706
**FCM**	CD11b	Mouse	Rat	M1/70	PerCP Cy5.5	1:100	BD Biosciences	550993
**FCM**	CD45	Mouse	Rat	30-F11	PeCy7	1:800	BD Biosciences	552848
**FCM**	CD206	Mouse	Rat	C068C2	APC	1:100	Biolegend	141708
**FCM**	CD11c	Mouse	Hamster	N418	APC	1:100	Biolegend	117310
**FCM**	Clec4f	Mouse	Mouse	3E3F9	Alexa Fluor 647	1:100	Biolegend	156804
**FCM**	Ly6G	Mouse	Rat	1A8	APC	1:200	Biolegend	127614
**FCM**	F4/80	Mouse	Rat	BM8	APC Cy7	1:100	Biolegend	123118
**FCM**	CD19	Mouse	Rat	6D5	APC Cy7	1:100	Biolegend	115529

Table S5. Patient demographics.

Characteristic	TNBC (n=4)	Melanoma (n=5)
Age		
Median	45	59
Range	34-75	55-60
Sex - no (%)		
Male	0 (0)	2 (40)
Female	4 (100)	3 (60)
Stage of Disease		
Stage III	0 (0)	0 (0)
Stage IV	4 (100)	5 (100)
Site of biopsy		
Liver	1	1
Lymph node	1	2
Neck	1	0
Chest wall	1	0
Abdomenal wall	0	1
Abdomenal	0	1
Response (RECIST v1.1)		
Complete Response (CR)	0	0
Partial Response (PR)	1	0
Stable Disease	3	2
Progressive Disease	0	3
