# Supplementary material for: Kupffer cells prevent pancreatic ductal adenocarcinoma metastasis to the liver in mice
Source: Nat Commun. 2023 Oct 10;14:6330. doi: 10.1038/s41467-023-41771-z (PMC10564762; doi:10.1038/s41467-023-41771-z)
Supplement: Supplementary file 3 — Reporting Summary [file 41467_2023_41771_MOESM3_ESM.pdf]

nature portfolio

Corresponding author(s): Max M. Wattenberg MD and Gregory L. Beatty MD, PhD.

Last updated by author(s): Aug 31, 2023

Reporting Summary

Nature Portfolio wishes to improve the reproducibility of the work that we publish. This form provides structure for consistency and transparency in reporting. For further information on Nature Portfolio policies, see our Editorial Policies and the Editorial Policy Checklist.

Statistics

For all statistical analyses, confirm that the following items are present in the figure legend, table legend, main text, or Methods section.

n/a	Confirmed	
☐	☒	The exact sample size (*n*) for each experimental group/condition, given as a discrete number and unit of measurement
☐	☒	A statement on whether measurements were taken from distinct samples or whether the same sample was measured repeatedly
☐	☒	The statistical test(s) used AND whether they are one- or two-sided<br>*Only common tests should be described solely by name; describe more complex techniques in the Methods section.*
☒	☐	A description of all covariates tested
☐	☒	A description of any assumptions or corrections, such as tests of normality and adjustment for multiple comparisons
☐	☒	A full description of the statistical parameters including central tendency (e.g. means) or other basic estimates (e.g. regression coefficient) AND variation (e.g. standard deviation) or associated estimates of uncertainty (e.g. confidence intervals)
☐	☒	For null hypothesis testing, the test statistic (e.g. *F*, *t*, *r*) with confidence intervals, effect sizes, degrees of freedom and *P* value noted<br>*Give P values as exact values whenever suitable.*
☒	☐	For Bayesian analysis, information on the choice of priors and Markov chain Monte Carlo settings
☒	☐	For hierarchical and complex designs, identification of the appropriate level for tests and full reporting of outcomes
☒	☐	Estimates of effect sizes (e.g. Cohen's *d*, Pearson's *r*), indicating how they were calculated

Our web collection on statistics for biologists contains articles on many of the points above.

Software and code

Policy information about availability of computer code

Data collection	No software was used for data collection. No custom algorithms were used and all relevant R packages are cited in the Methods.
Data analysis	Software used for data analysis is described in the Materials and Methods section. Briefly, immunohistochemistry analysis was conducted using Visiopharm software (version 2020.01.1.7332). Immunofluorescence microscopy specimen were anlayzed using Tissue Finder software and Flowjo (version 10.8). Flow cytometry data was analyzed using Flowjo (version 10.8). Single cell RNA seq analysis was conducted using Seurat (version 4.1.1.). Statistical analysis was performed in R and using Prism (version 9.2.0). Pseudobulk differentially expressed gene analysis was performed using DESeq2. Radar plots were generated using the radarchart R package.

For manuscripts utilizing custom algorithms or software that are central to the research but not yet described in published literature, software must be made available to editors and reviewers. We strongly encourage code deposition in a community repository (e.g. GitHub). See the Nature Portfolio guidelines for submitting code & software for further information.

Data

Policy information about availability of data

All manuscripts must include a data availability statement. This statement should provide the following information, where applicable:

- Accession codes, unique identifiers, or web links for publicly available datasets
- A description of any restrictions on data availability
- For clinical datasets or third party data, please ensure that the statement adheres to our policy

Source data are provided for this paper. Raw and processed scRNAseq files are available from Gene Expression Omnibus (GEO) under accession numbers

GSE235318 [https://www.ncbi.nlm.nih.gov/geo/query/acc.cgi?acc=GSE235318]. The mm10 transcriptome is available at https://www.ncbi.nlm.nih.gov/datasets/genome/GCF_000001635.20/

Research involving human participants, their data, or biological material

Policy information about studies with human participants or human data. See also policy information about sex, gender (identity/presentation), and sexual orientation and race, ethnicity and racism.

Reporting on sex and gender	No patients were excluded based on sex or gender. Sex was self-reported. Both male and female patients were recruited. All patients with breast cancer (n=4) were female. Patients with melanoma were male (n=2) and female (n=3). Sample sizes of human participants precluded sex-based subset analysis. Patient characteristics are included in Supplementary Table S5.
Reporting on race, ethnicity, or other socially relevant groupings	No patients were excluded based on race, ethnicity or other socially relevant groupings. Further, given the sample size of the included data sets, these factors were not incorporated into the presented analysis.
Population characteristics	Age, sex, disease stage, site of biopsy and radiologic response were abstracted from the clinical database (Supplementary Table S5). Given the small sample size, it remains possible that confounding introduced by these covariates could influence the analysis.
Recruitment	Source of human materials is detailed in the Materials and Methods. Biopsy samples from human PDAC liver metastasis lesions were collected from patients enrolled in the Pancreatic Cancer Action Network (PanCAN) Know Your Tumor (KYT) program. For this program, patients self-refer to the PanCAN call center and enroll through an institutional review board (IRB) approved protocol. Paired biopsies from non-targeted lesions from liver (n=2), lymph node (n=4), neck (n=1), chest wall (n=1), and abdomen (n=1) were collected from patients enrolled on the IMPRIME-1 trial (NCT02981303), a multicenter, open-label, Phase 2 study of soluble β-glucan (odetiglucan) and pembrolizumab (KEYTRUDA®, pembrolizumab).
Ethics oversight	The Pancreatic Cancer Action Network (PanCAN) Know Your Tumor (KYT) study was approved by the New England IRB. The IMPRIME-1 trial was approved by central or local IRBs including Western Institutional Review Board, UCLA Office of Human Research Protection Program and Mayo Clinic Institutional Review Board. Research was conducted in accordance with recognized ethical guidelines including the Belmont Report, CIOMS, Declaration of Helsinki, and U.S. Common Rule.

Note that full information on the approval of the study protocol must also be provided in the manuscript.

Field-specific reporting

Please select the one below that is the best fit for your research. If you are not sure, read the appropriate sections before making your selection.

- [x] Life sciences
- [ ] Behavioural & social sciences
- [ ] Ecological, evolutionary & environmental sciences

For a reference copy of the document with all sections, see nature.com/documents/nr-reporting-summary-flat.pdf

Life sciences study design

All studies must disclose on these points even when the disclosure is negative.

Sample size	Sample sizes were estimated based on pilot experiments conducted in the laboratory and were selected to provide sufficient numbers of mice in each group to yield a two-sided statistical test, with the potential to reject the null hypothesis with a power of (1-beta) of 80%.
Data exclusions	Mice that were euthanized early due to health concerns or surgical complications were excluded from studies as pre-established by the University of Pennsylvania IACUC protocol #803605. Samples that did not stain properly after two attempts of automated IHC staining were excluded. ROUT or Grubb outliers test was applied to analysis of IHC images and identified outliers excluded.
Replication	Data are representative of at least two independent experiments unless otherwise noted in the figure legends.
Randomization	For all studies, mice of similar age and sex were used. Both male and female mice between 8 to 12 weeks of age were used, except for overall survival and scRNAseq studies where only female mice were used. Mice were enrolled in a randomized, unblinded fashion. For studies involving human specimens analysis was performed on tissues grouped by tumor type and comparisons made between pre- and post-treatment specimens or between cell types within the same tissue.
Blinding	Blinding was not possible due to obvious gross effects of therapy and as research personnel required knowledge of the individual experimental groups. However, findings were validated in multiple biological replicates and independent models.

Reporting for specific materials, systems and methods

We require information from authors about some types of materials, experimental systems and methods used in many studies. Here, indicate whether each material, system or method listed is relevant to your study. If you are not sure if a list item applies to your research, read the appropriate section before selecting a response.

Materials & experimental systems

n/a	Involved in the study
☐	☒ Antibodies
☐	☒ Eukaryotic cell lines
☒	☐ Palaeontology and archaeology
☐	☒ Animals and other organisms
☐	☒ Clinical data
☒	☐ Dual use research of concern
☒	☐ Plants

Methods

n/a	Involved in the study
☒	☐ ChIP-seq
☐	☒ Flow cytometry
☒	☐ MRI-based neuroimaging

Antibodies

Antibodies used

Antibodies are detailed in Materials and Methods and Supplementary Tables S1-4. PD-1, Bio X Cell, RMP1-14, BE0146; CD4, Bio X Cell, GK1.5, BP0003-1; CD8, Bio X Cell, 2.43, BE0061; Rat IgG2b isotype, Bio X Cell, LFT-2, BE0090; Clec4f, R&D, polyclonal, AF2784; GFP, Abcam, Polyclonal, ab6673; CD3, Abcam, Polyclonal, ab5690; F4/80, Cell Signaling, D2S9R, 70076; Ki67, Cell Signaling, D3B5, 12202; CK19, Abcam, EPNCIR127B, ab133496; CD68, Roche, Kp1, 790-2931; Ki67, Roche, 30-9, 790-4286; CK19, Roche, A53-B/A2.26, 760-4281; Foxp3, Abcam, 236A/E7, Ab20034; Cytokeratin-Opal 480 or 780, Dako, AE1/AE3, M515; CD8a-Opal 520, Thermo, AMC908, 14-008-82; Granzyme B-Opal 570, Abcam, Polyclonal, 14-008-82; FoxP3-Opal 620, Thermo, eBio7979, 14-7979-82; CD4-Opal 690, Thermo, N1UG0, 14-2444-82; Ki67-Opal 780, Thermo, Sp6, MA5-14520; Melanoma triple cocktail-Opal 480 or 780, Ventana, Polyclonal, 790-4677; PD-L1-Opal 620, Cell Signaling, E1L3N, 13684; CD163-Opal 570, Abcam, EPR19518, 182422; CD80-Opal 520, Genetex, 2E5, GTX84700; CD68-Opal 480, Abcam, Kp1, 955; CD206-Opal 690, Abcam, Polyclonal, 64693; Anti-rabbit-HRP, Jackson Immunoresearch, Polyclonal, 111-035-144; Anti-mouse-HRP, Jackson Immunoresearch, Polyclonal, 115-035-146; CD3-Pac blue, Biolegend, 17A2, 100214; Ly6G-Pac blue, Biolegend, 1A8, 127612; Ly6G-BV421, Biolegend, 1A8, 127627; CD11c-Pac blue, Biolegend, N418, 117322; CD19-Pac blue, Biolegend, 6D5, 115523; CD206-FITC, Biolegend, C068C2, 141704; CD38-FTIC, Biolegend, 90, 102705; CD19-FITC, Biolegend, 6D5, 115506; Dectin1-PE, Biolegend, Rh1, 144303; H-2Kb/H-2Db-PE, Biolegend, 28-8-6, 114607; CD38-PE, Biolegend, 90, 102708, CD11c, Biolegend, HL3, 553802; CD206-PE, Biolegend, C068C2, 141706; CD11b-PerCP Cy5.5, BD Bioscences, M1/70, 550993; CD45-PeCy7, BD Biosciences, 30-F11, 552848; CD206-APC, Biolegend, C068C2, 141708; CD11c-APC, Biolegend, N418, 117310; Clec4f-Alexa Fluor 647, Biolegend, 3E3F9, 156804; Ly6G-APC, Biolegend, 1A8, 127614; F4/80-APC Cy7, Biolegend, BM8, 123118; CD19-APC Cy7, Biolegend, 6D5, 115529.

Validation

Antibodies are detailed in Materials and Methods and Supplementary Tables S1-4. Each antibody used in this study was validated by its manufacturer.
PD-1, Bio X Cell, RMP1-14, BE0146, https://bioxcell.com/invivomab-anti-mouse-pd-1-cd279-be0146;
CD4, Bio X Cell, GK1.5, BP0003-1, https://bioxcell.com/invivoplus-anti-mouse-cd4-bp0003-1;
CD8, Bio X Cell, 2.43, BE0061, https://bioxcell.com/invivomab-anti-mouse-cd8a-be0061;
Rat IgG2b isotype, Bio X Cell, LFT-2, BE0090, https://bioxcell.com/invivomab-rat-igg2b-isotype-control-anti-keyhole-limpet-hemocyanin-be0090;
Clec4f, R&D, polyclonal, AF2784, https://www.rndsystems.com/products/mouse-clec4f-clecsf13-antibody_af2784?gclid=CjwKCAjwloynBhBbEiwAGY25dANwa2qq3qGp8iooi02gTCe6uHjPr7uSHhmQuicYVqmeTVFQk36wWRoCJyMQAvD_BwE&gclsrc=aw.ds;
GFP, Abcam, Polyclonal, ab6673, https://www.abcam.com/products/primary-antibodies/gfp-antibody-ab6673.html;
CD3, Abcam, Polyclonal, ab5690, https://www.abcam.com/products/primary-antibodies/cd3-antibody-ab5690.html;
F4/80, Cell Signaling, D2S9R, 70076, https://www.cellsignal.com/products/primary-antibodies/f4-80-d2s9r-xp-rabbit-mab/70076;
Ki67, Cell Signaling, D3B5, 12202, https://www.cellsignal.com/products/primary-antibodies/ki-67-d3b5-rabbit-mab-ihc-formulated/12202;
CK19, Abcam, EPNCIR127B, ab133496, https://www.abcam.com/products/primary-antibodies/cytokeratin-19-antibody-epncir127b-ab133496.html;
CD68, Roche, Kp1, 790-2931, https://diagnostics.roche.com/global/en/products/lab/cd68-kp-1-confirm-rtd000775.html;
Ki67, Roche, 30-9, 790-4286, https://diagnostics.roche.com/global/en/products/lab/ki-67-30-9-confirm-rtd000792.html;
CK19, Roche, A53-B/A2.26, 760-4281, https://diagnostics.roche.com/global/en/products/lab/cytokeratin-19-a53-b-a2-26-rtd000821.html;
Foxp3, Abcam, 236A/E7, Ab20034, https://www.abcam.com/products/primary-antibodies/foxp3-antibody-236ae7-ab20034.html;
Cytokeratin, Dako, AE1/AE3, M515, https://www.agilent.com/en/product/immunohistochemistry/antibodies-controls/primary-antibodies/cytokeratin-(concentrate)-76562;
CD8a, Thermo, AMC908, 14-0008-82, https://www.thermofisher.com/antibody/product/CD8a-Antibody-clone-AMC908-Monoclonal/14-0008-82;
Granzyme B, Abcam, Polyclonal, 4059, https://www.abcam.com/products/primary-antibodies/granzyme-b-antibody-ab4059.html;
FoxP3, Thermo, eBio7979, 14-7979-82, https://www.thermofisher.com/antibody/product/FOXP3-Antibody-clone-eBio7979-221D-D3-Monoclonal/14-7979-82;
CD4-, Thermo, N1UG0, 14-2444-82, https://www.thermofisher.com/antibody/product/CD4-Antibody-clone-N1UG0-Monoclonal/14-2444-82;
Ki67, Thermo, Sp6, MA5-14520, https://www.thermofisher.com/antibody/product/Ki-67-Antibody-clone-SP6-Recombinant-Monoclonal/MA5-14520;
Melanoma triple cocktail, Ventana, Polyclonal, 790-4677, https://diagnostics.roche.com/global/en/products/lab/melanoma-triple-cocktail-hmb45-a103-t311-rtd001037.html;
PD-L1, Cell Signaling, E1L3N, 13684, https://www.cellsignal.com/products/primary-antibodies/pd-l1-e1l3n-xp-rabbit-mab/13684;
CD163, Abcam, EPR19518, 182422, https://www.abcam.com/products/primary-antibodies/cd163-antibody-epr19518-

ab182422.html;
CD80, Genetex, 2E5, GTX84700, https://www.genetex.com/Product/Detail/CD80-antibody-2E5/GTX84700;
CD68, Abcam, Kp1, 955, https://www.abcam.com/products/primary-antibodies/cd68-antibody-kp1-ab955.html;
CD206, Abcam, Polyclonal, 64693, https://www.abcam.com/products/primary-antibodies/mannose-receptor-antibody-ab64693.html;
Anti-rabbit-HRP, Jackson Immunoresearch, Polyclonal, 111-035-144, https://www.jacksonimmuno.com/catalog/products/111-035-144;
Anti-mouse-HRP, Jackson Immunoresearch, Polyclonal, 115-035-146, https://www.jacksonimmuno.com/catalog/products/115-035-146;
CD3-Pac blue, Biolegend, 17A2, 100214, https://www.biolegend.com/en-gb/products/pacific-blue-anti-mouse-cd3-antibody-3317;
Ly6G-Pac blue, Biolegend, 1A8, 127612, https://www.biolegend.com/en-gb/products/pacific-blue-anti-mouse-ly-6g-antibody-6082;
Ly6G-BV421, Biolegend, 1A8, 127627, https://www.biolegend.com/en-gb/products/brilliant-violet-421-anti-mouse-ly-6g-antibody-7161;
CD11c-Pac blue, Biolegend, N418, 117322, https://www.biolegend.com/en-gb/products/pacific-blue-anti-mouse-cd11c-antibody-3864;
CD19-Pac blue, Biolegend, 6D5, 115523, https://www.biolegend.com/en-gb/products/pacific-blue-anti-mouse-cd19-antibody-2987;
CD206-FITC, Biolegend, C068C2, 141704, https://www.biolegend.com/en-gb/products/fitc-anti-mouse-cd206-mmr-antibody-7318;
CD38-FTIC, Biolegend, 90, 102705, https://www.biolegend.com/en-gb/products/fitc-anti-mouse-cd38-antibody-182;
CD19-FITC, Biolegend, 6D5, 115506, https://www.biolegend.com/en-gb/products/fitc-anti-mouse-cd19-antibody-1528;
Dectin1-PE, Biolegend, Rh1, 144303, https://www.biolegend.com/en-gb/products/pe-anti-mouse-cd369-dectin-1-clec7a-antibody-8102;
H-2Kb/H-2Db-PE, Biolegend, 28-8-6, 114607, https://www.biolegend.com/en-gb/products/pe-anti-mouse-h-2k-b-h-2d-b-antibody-1686;
CD38-PE, Biolegend, 90, 102708, CD11c, Biolegend, HL3, 553802, https://www.biolegend.com/en-gb/search-results?Keywords=+553802;
CD206-PE, Biolegend, C068C2, 141706, https://www.biolegend.com/en-gb/search-results?Keywords=+141706;
CD11b-PerCP Cy5.5, BD Biosciences, M1/70, 550993, https://www.bdbiosciences.com/en-us/products/reagents/flow-cytometry-reagents/research-reagents/single-color-antibodies-ruo/percp-cy-5-5-rat-anti-cd11b.550993;
CD45-PeCy7, BD Biosciences, 30-F11, 552848, https://www.bdbiosciences.com/en-us/products/reagents/flow-cytometry-reagents/research-reagents/single-color-antibodies-ruo/pe-cy-7-rat-anti-mouse-cd45.552848;
CD206-APC, Biolegend, C068C2, 141708, https://www.biolegend.com/en-gb/products/apc-anti-mouse-cd206-mmr-antibody-7425;
CD11c-APC, Biolegend, N418, 117310, https://www.biolegend.com/en-gb/products/apc-anti-mouse-cd11c-antibody-1813;
Clec4f-Alexa Fluor 647, Biolegend, 3E3F9, 156804, https://www.biolegend.com/en-gb/explore-new-products/alexa-fluor-647-anti-mouse-clec4f-antibody-18329?GroupID=GROUP20;
Ly6G-APC, Biolegend, 1A8, 127614; https://www.biolegend.com/en-gb/products/apc-anti-mouse-ly-6g-antibody-6115
F4/80-APC Cy7, Biolegend, BM8, 123118, https://www.biolegend.com/en-gb/products/apc-cyanine7-anti-mouse-f4-80-antibody-4072;
CD19-APC Cy7, Biolegend, 6D5, 115529, https://www.biolegend.com/en-gb/products/apc-cyanine7-anti-mouse-cd19-antibody-3903.

Eukaryotic cell lines

Policy information about cell lines and Sex and Gender in Research

Cell line source(s)	PDA.8572 (PDAC–YFP) and PDA.69 (PDAC.69) cell lines were used for intrasplenic, intraportal, and tail-vein injections. PDA.8572 cell line was derived from a PDAC tumor that arose spontaneously in a female KPCY mouse, as previously described[52, 59, 75]. PDA.69 was derived from a PDAC tumor that arose spontaneously in a female KPC mouse, as previously described [52].
Authentication	Cell lines were previously established as described and no further authentication was performed.
Mycoplasma contamination	Cell lines were tested routinely for Mycoplasma contamination at the Cell Center Services Facility at the University of Pennsylvania and all lines used tested negative for contamination.
Commonly misidentified lines (See ICLAC register)	No commonly misidentified cell lines were used.

Animals and other research organisms

Policy information about studies involving animals; ARRIVE guidelines recommended for reporting animal research, and Sex and Gender in Research

Laboratory animals	C57BL/6J, Clec4f-cre (C57BL/6J-Clec4fem1(cre)Glass/J) and Rosa-DTR (C57BL/6-Gt(ROSA)26Sortm1(HBEGF)Awai/J) mice were procured from the Jackson Laboratory. Clec4f-cre+/+ mice were crossed with Rosa-DTRflox/flox to generate Clec4fDTR (Clec4f-cre+/- Rosa-DTRflox/+) mice for use in experiments. Mice of all genotypes were used at ages between 8 and 12 weeks.
Wild animals	No wild animals were used.
Reporting on sex	For all studies, mice of similar age and sex were used. Both male and female mice between 8 to 12 weeks of age were used, except for overall survival and scRNAseq studies where only female mice were used.

Field-collected samples	No field-collected samples were used.
Ethics oversight	All mice were bred and maintained in the animal facility of the University of Pennsylvania. Animal protocols were reviewed and approved by the Institute of Animal Care and Use Committee of the University of Pennsylvania.

Note that full information on the approval of the study protocol must also be provided in the manuscript.

Clinical data

Policy information about clinical studies

All manuscripts should comply with the ICMJE guidelines for publication of clinical research and a completed CONSORT checklist must be included with all submissions.

Clinical trial registration	NCT02981303
Study protocol	Protocol details are available at https://clinicaltrials.gov/ct2/show/NCT02981303
Data collection	Data was collected between December 5, 2016 and March 25, 2021.
Outcomes	An exploratory analysis of pre- and post-treatment tumor biopsies using microscopy was performed.

Flow Cytometry

Plots

Confirm that:

- [x] The axis labels state the marker and fluorochrome used (e.g. CD4-FITC).
- [x] The axis scales are clearly visible. Include numbers along axes only for bottom left plot of group (a 'group' is an analysis of identical markers).
- [x] All plots are contour plots with outliers or pseudocolor plots.
- [x] A numerical value for number of cells or percentage (with statistics) is provided.

Methodology

Sample preparation	For experiments assessing tumor burden, mice were euthanized and then the portal vein and inferior vena cava were severed to drain the blood from the liver and lung. For experiments assessing resident immune cell phenotype, mice were euthanized and then the lung and liver were perfused with PBS through the portal vein, inferior vena cava and the heart. Successful perfusion was achieved by complete blanching of the organ. Liver and lung were removed and rinsed in DMEM complete media and then minced with micro-dissecting scissors into small pieces in DMEM containing collagenase (1 mg/ml, Sigma-Aldrich) and DNase (150 U/ml, Roche). Tissues were then incubated at 37°C for 30 min with intermittent agitation, filtered through a 70µm nylon strainer (Corning), and washed three times with FACS buffer (PBS with 2% FBS and 0.2mM EDTA). Lysis of red blood cells was performed using ACK lysing buffer (Quality Biological) at room temperature for 5 minutes. Cells were then washed with FACS buffer. Cell suspension was then passed through a 40µm strainer. Peripheral blood was collected from the tail vein of mice into a capillary tube. Red blood cells were lysed in ACK lysis buffer. Cells were then counted using a BioRad TC20 automated cell counter and stained using Aqua dead cell stain kit (Life Technologies) according to the manufacturer's protocol. For immune cell subset characterization, cells were washed twice with FACS buffer and stained with appropriate antibodies.
Instrument	Samples were examined using a FACS Canto II (BD Biosciences).
Software	Analysis was performed using Flowjo (FlowJo, LLC, version 10.8).
Cell population abundance	The abundance of relevant cell populations was determined by normalization to organ/tissue weight and represented in the main figures and supplementary materials. Cell purity was determined using relevant lineage markers for tumor and immune cell types.
Gating strategy	Gating was performed to exclude debris and event doublets by comparing FSC and SSC parameters. Dead cells were excluded using Aqua dead cell stain kit (Life Technologies). Fluorescent minus one controls were used to define gating boundaries.

- [x] Tick this box to confirm that a figure exemplifying the gating strategy is provided in the Supplementary Information.
